# The Effect of Students’ Perception of Teachers’ Emotional Support on School Burnout Dimensions: Longitudinal Findings

**DOI:** 10.3390/ijerph18041922

**Published:** 2021-02-17

**Authors:** Luciano Romano, Giacomo Angelini, Piermarco Consiglio, Caterina Fiorilli

**Affiliations:** Department of Human Sciences, University of Rome LUMSA, 00193 Rome, Italy; g.angelini@lumsa.it (G.A.); piermarco.consiglio@gmail.com (P.C.); fiorilli@lumsa.it (C.F.)

**Keywords:** school burnout, high school, students’ perception, teachers’ emotional support

## Abstract

School burnout is linked to relevant adverse consequences for students’ academic careers. Thus, several authors have focused on the internal and external factors that reduce burnout, highlighting the role of teachers’ support. Nonetheless, few studies addressed how students’ perception of teachers’ emotional support protects them from school maladaptive behaviors. The present study aimed to longitudinally investigate in a final sample of 295 Italian high school students (F = 78.6%; M = 15.78, SD = 1.48) the protective role of students’ perception of teachers’ emotional support dimensions on school burnout across a school year. We expected that teachers’ emotional support dimensions had a significant inverse effect on students’ burnout. We preliminarily investigated the study variables’ associations and whether the mean levels of burnout dimensions increased throughout the school year. Correlation analysis supported the associations among the study variables, and repeated-measures analysis of variance (ANOVA) analyses highlighted that the mean levels of school burnout dimensions increased over time. Moreover, hierarchical multiple regression analyses have shown that at the beginning of the school year (T1), the teacher sensitivity dimension significantly and inversely affected emotional exhaustion by the end of the school year (T2). Our findings shed light on the role played by teacher emotional support and give suggestions on which specific facet should have to be improved to shield students from later burnout-related exhaustion.

## 1. Introduction

School burnout is a syndrome of physical, cognitive, and emotional exhaustion due to a chronic over-exposure to high levels of academic stress [1]. Usually, school burnout is composed of three dimensions, i.e., emotional exhaustion, a cynical attitude toward school, and feelings of inadequacy as a student [1,2,3]. Specifically, emotional exhaustion refers to a sense of chronic fatigue and being overwhelmed by school-related pressures. Recent studies have considered emotional exhaustion as the core dimension and first sign of this syndrome (e.g., [4,5,6]). Cynicism concerns a disinterested and demotivated attitude toward the academic context. Several authors posited that it represents a coping strategy to counteract and distance from the burnout-related emotional exhaustion, predicting the subsequent feeling of inadequacy [4,6,7,8]. Finally, inadequacy as a student pertains to the sensation of being useless and inefficient toward their role, with a reduced feeling of competence, personal satisfaction, and sense of belonging [1,7,9,10]. Although previous studies highlighted that the three burnout dimensions overlap, further studies have demonstrated that they are separate but closely linked constructs [4,11].

Previous studies have underlined the detrimental consequences of burnout for adolescents, both inside (e.g., low academic performance, school dropout) and outside (e.g., later depression) of the school context [12,13,14,15,16]. Moreover, evidence suggested that school burnout symptoms increase across students’ career due to a growing imbalance between external demands and available students’ resources (e.g., [17]). Students indeed have to deal with several new challenges during high school, such as daily tests and examinations, which are utterly different from those they have learned to deal with in middle school [18]. Besides, adolescents are also expected to be high performers during their school years. For instance, they know right away that they have to study hard to prepare for the final examination at the end of their track, which is often crucial for being admitted to high-profile universities (e.g., [19]). This kind of continuous and taxing pressure could lead some pupils to experience several emotional and academic maladjustments (e.g., [20,21]).

Nonetheless, when students may count on an adequate amount of resources, both personal (e.g., self-esteem) and external (i.e., social support), they can also resist the overwhelming emotional burden related to excessive school demands. In other words, resources represent the protective equipment against students’ burnout-related consumption in the long term (e.g., [22]). In detail, and focusing on external resources, students in a highly supportive environment are more prone to communicate their worries and difficulties, as far as they know that close others would help them. When feeling loved and supported, students perceive that they have secure bonds, which, in turn, helps them to shape the impact of adverse life events, including negative school results [23,24]. Previous studies have highlighted the pivotal role of teacher support in promoting students’ well-being and overall adjustment, both in comprehensive and upper secondary schools (e.g., [11,25,26,27]). According to Hughes and colleagues [26], students’ perception of teacher support was more related to students’ well-being and dedication toward the school than other support sources (e.g., family and friends). Overall, students cope with negative school life-events depending on whether they perceive teachers’ support. The more teachers are attentive toward their worries at the beginning of the school year, the more students’ well-being increases [17,22].

Despite the abovementioned evidence, it remains less investigated the protective role of teachers’ emotional support towards school burnout in a longitudinal perspective.

Teachers’ emotional support pertains to teachers’ ability in showing caring behaviors towards students’ emotional and social needs, and encompasses three dimensions, namely positive climate (i.e., a teacher’s positive attitudes towards students), teacher sensitivity (i.e., teacher responsiveness to students’ academics, emotional abilities, and needs), and regard for the adolescent perspective (i.e., a teacher’s ability to support students and promote their development) [28,29]. To our knowledge, two previous studies addressed this issue, showing that perceived teacher emotional support and its dimensions were significantly and negatively related to high school students’ burnout. However, both studies used the cross-sectional approach [19,30].

Thus, the current study’s central core was to examine students’ burnout levels at the beginning (time 1, T1) and at the end (time 2, T2) of the school year by analyzing the impact of teachers’ emotional support. In this regard, we set three hypotheses. First, we investigated associations among the study’s variables at T1 and T2. We expected negative correlations in line with existing literature: the more students perceive a high level of teachers’ emotional support, the less they feel burned out. Simultaneously and coherently with several studies, we expected that exhaustion, cynicism, and sense of inadequacy were all significantly and positively correlated with each other both at T1 and T2 (H1). As the second hypothesis, we expected that the levels of emotional exhaustion, cynicism, and sense of inadequacy as a student would increase from the beginning (T1) to the end of the school year (T2) (H2). Finally, we aimed to investigate whether and to what extent perceived emotional support from teachers affects students’ burnout. We expected that the dimensions of perceived teacher emotional support at T1 would negatively affect school burnout dimensions at T2 (H3).

## 2. Materials and Methods

### 2.1. Participants and Procedure

The current study was carried out in Italy during the school year 2018/2019 with data collection at two time points. Administrations at Time 1 took place at the beginning of the school year (October 2018, T1), and the ones at Time 2 occurred at the end of the school year (June 2019, T2). At T1, 404 Italian high school students (83.7 % female, M = 15.85, SD = 1.61) took part in the study; among the whole sample, a total of 295 (78.6% female), aged between 13 and 19 years (M = 15.78, SD = 1.48), took part in the study at T2. Only the students who participated in both data collection sections (T1 and T2) were included in the final sample (the participation rate was 73%). Students (at T2) belonged to two different public high schools in central (88.5%) and southern Italy (11.5%). Specifically, 77.6% of them attended a human sciences high school (85.8% female), and 22.4% attended a high school specializing in classics subjects (30.2% female). Students completed a self-report questionnaire with a paper–pencil approach. Furthermore, they inserted a personal and anonymized identification number needed to match the questionnaires answered in the data collection at the two time points. The administrations were conducted during regular school hours. A member of the research team was present to provide all the necessary information to complete the questionnaire. The administration procedure lasted approximately 30 minutes. Teachers were asked to leave the classroom while students completed the questionnaires. Participation was voluntary, and anonymity and confidentiality standards were assured for all participants. Furthermore, only students of age who provided written informed consent, and only underage students with a signed consent form from their parents, could take part in the study. The school council approved the research before the administrations. All the study procedures were in accordance with the Declaration of Helsinki of 1964 and its latest version. The Ethics Committee of the Lumsa University of Rome, Italy approved the research protocol.

### 2.2. Instruments

Perceived teachers’ emotional support. Participants answered items of the Teacher Emotional Support Scale Italian version (TESS) [30]. The TESS is composed of 15 items on a 5-point Likert scale (1 = “Not at all true”, 5 = “Very true”) and comprises three dimensions: Positive climate, Teacher sensitivity, and Regard for adolescent perspective. Specifically, and concerning the measurements at T1, Cronbach’s alpha was 0.90 for the total score, 0.73 for Positive climate, 0.90 for Teacher sensitivity, and 0.79 for Regard for adolescent perspective. At T2, Cronbach’s alpha was 0.93 for the total score, 0.82 for Positive climate, 0.89 for Teacher sensitivity, and 0.82 for Regard for adolescent perspective.

School Burnout. Students answered items of the School Burnout Inventory Italian version (SBI) [2]. The SBI is composed of 9 items on a 6-point Likert scale (1 = “I totally disagree”, 6 = “I totally agree”) and comprises three subscales: Emotional exhaustion, Cynicism, and Sense of inadequacy. Cronbach’s alpha at T1 was 0.85 for the total score, 0.76 for Emotional exhaustion, 0.80 for Cynicism, and 0.60 for Sense of inadequacy. At T2, Cronbach’s alpha was 0.86 for the total score, 0.78 for Emotional exhaustion, 0.82 for Cynicism, and 0.69 for Sense of inadequacy.

### 2.3. Analysis Plan

Firstly, and to verify H1, we obtained means and standard deviations and performed a correlation analysis among the study variables.

Then, we performed repeated-measures ANOVA analyses to verify H2, comparing the mean at T1 vs. T2 for the three school burnout dimensions.

Finally, three stepwise hierarchical multiple regressions verified H3, one for each burnout dimension (outcome). In detail, firstly, we controlled for age and the corresponding output at T1. Secondly, we added the three dimensions of students’ perception of teachers’ emotional support at T1. All the statistical analyses were conducted on the final sample of 295 students who participated in data collection at both time points.

## 3. Results

Table 1 reports the means, standard deviations, and correlations. The sex variable was treated as a dummy variable, where 0 was the value attributed to females and 1 was the value attributed to males.

The correlation analysis revealed that both at T1 and T2 perceived teacher emotional support dimensions were significantly and negatively associated with school burnout dimensions (*p* < 0.01). Moreover, the three school burnout dimensions were significantly related to each other both at T1 and T2 (*p* < 0.01), as expected.

Repeated measures ANOVAs highlighted a significant effect of time on Emotional exhaustion (F(1, 294) = 73.72, *p* = 0.000), Cynicism (F(1, 294) = 54.52, *p* = 0.000), and Sense of inadequacy (F(1, 294) = 56.90, *p* = 0.000). The mean levels of all the school burnout dimensions significantly increased from T1 to T2 (Table 1).

Results from the three stepwise hierarchical multiple regressions (Table 2) show that only Teacher sensitivity at T1 significantly and negatively predicted Emotional exhaustion at T2 (β = −25, t = −3.82, *p* < 0.01), and the overall model longitudinally accounted for 46% of its variance. Although Regard for adolescent perspective at T1 significantly and negatively predicted Sense of Inadequacy at T2 (β = −15, t = −2.25, *p* < 0.05), the change in R^2^ from Step 1 to Step 2 was not significant (*p* > 0.05).

## 4. Discussion

The current study starts with the awareness that adolescents are particularly exposed to burnout risk. Several findings widely support the increase in the school burnout dimensions’ levels (i.e., emotional exhaustion, cynicism, and sense of inadequacy) as students move forward into the school year [5,31]. In this regard, existing findings show that high school students have a high risk of disengagement and dropout due to the imbalance between personal resources and school demands [12,17], leading to the school burnout experience. At the same time, external resources may significantly and positively influence students’ well-being, reducing the adoption of negative coping strategies like cynicism [32]. Our study addresses this topic in a longitudinal perspective by investigating the impact of teachers’ emotional support (i.e., as external support perceived by the student) during one school year in a sample of Italian high school students. Overall, findings support the study hypotheses, as discussed below.

Firstly, and coherent with prior findings (e.g., [30]), the teachers’ emotional support dimensions maintain negative correlations with school burnout subscales at the beginning and the end of the school year. Besides, students’ emotional exhaustion is positively associated with cynicism and a sense of inadequacy, both at the beginning and the end of the school year. Extensive and international findings support the Italian results, which highlight that being a burned-out student regards simultaneously an emotional state (feeling exhausted and inadequate), and a coping strategy (adopting cynicism) (e.g., [4,6,10]). As expected, our findings also confirmed, on the one hand, the negative associations between students’ perception of emotional support, and, on the other, the school burnout dimensions. Previous studies support our hypothesis regarding external resources’ positive role in students’ adaptation to their school life [33]. Effectively, the more they feel supported, the less they risk burnout syndrome.

Secondly, the present findings support our expectation to observe an increase in students’ burnout levels from T1 to T2, consistent with the other countries’ results [4,5,14]. Several scholars have analyzed why burnout rises during the teenage period. According to the existing literature (e.g., [34]), the period of adolescence is critical to new developmental tasks. Students perceive several requirements arising from their social group of belonging (e.g., physical, psychological, and social). Effectively, young people perceive more pressure to achieve school-related competencies, on the one hand, and homework overload, on the other one. Combining this with the high risk of emotional disorders during adolescence (e.g., [35]), it is not surprising that school burnout may arise during adolescence-age life. Moreover, in the current study, we observed an increasing level of burnout in less than twelve months, which should draw teachers’ attention to students’ well-being more carefully. Recent evidence from the Organisation for Economic Co-operation and Development’s survey (OECD) [36] highlights that a positive student–teacher relationship boosts students’ school adjustment and well-being.

In this regard, approaching our main results, we observe the beneficial role of teachers’ emotional support toward students’ burnout development trajectory from T1 to T2. In detail, the findings partially confirm our last hypothesis, showing that only the teacher sensitivity dimension at T1 inversely affected emotional exhaustion at T2 (for example, students agree with items like “Our teachers care about how we feel”). In other words, the more teachers were responsive and empathic with students’ emotional needs and worries, the less students’ exhaustion occurs during the school year.

Interestingly, existing studies highlight that even though adolescent students ask for self-determination to manage school-life events, they need to count on positive support from others (with teachers as the most crucial support source) [17,22,37]. Coherently, further studies referring to the Italian context show that teachers’ emotional support is an essential source for students’ school transitions (e.g., [38]).

The present study yields suggestions (for teachers, practitioners, and policymakers) on the empowerment of a specific facet of the relations settled with students at the beginning of the school year, such as teacher sensitivity.

Two main limitations need to be considered for future research: a short observation period and a unique burnout measure. Future studies should also adopt a multi-informant design involving teachers and students to analyze their reciprocal relationship deeply.

## 5. Conclusions

Burnout represents a serious and current issue in school maladjustment that could not be disregarded. In line with international literature in this field, our study has also shown that the mean levels of school burnout dimensions tend to rise throughout the school year in Italian high school students. Nevertheless, the protective role of teachers’ sensitivity may reduce burnout risk. This result sheds light on the importance of strengthening the teacher–student emotional relationship. If teachers manage to enhance their emotional closeness early on, their students would be more shielded against future burnout-related exhaustion. When a student feels overwhelmed and exhausted due to school demands, the perception of emotional closeness with his/her teacher may serve as external support by reducing the solution-focused self-help approach. Overall, teachers should pay more attention to their students’ emotional states and lead them to face their school tasks efficiently.

## Figures and Tables

**Table 1:** Descriptive statistics and correlation matrix.

Table	M	SD	1	2	3	4	5	6	7	8	9	10	11	12	13
Time 1															
1. SEX															
2. AGE	15.78	1.48	−0.098												
3. EXH	11.58	4.71	−0.123 *	0.244 **											
4. CYN	7.40	4.04	0.001	0.222 **	0.465 **										
5. INAD	5.13	2.62	0.002	0.204 **	0.503 **	0.692 **									
6. PC	18.72	3.91	0.019	−0.374 **	−0.298 **	−0.260 **	−0.258 **								
7. TS	17.23	5.56	0.075	−0.372 **	−0.390 **	−0.329 **	−0.370 **	0.558 **							
8. RAP	11.79	3.57	0.046	−0.272 **	−0.267 **	−0.296 **	−0.305 **	0.504 **	0.694 **						
Time 2															
9. EXH	13.61	5.04	−0.213 **	0.226 **	0.657 **	0.284 **	0.337 **	−0.240 **	−0.406 **	−0.226 **					
10. CYN	8.94	4.33	0.001	0.227 **	0.310 **	0.637 **	0.520 **	−00.271 **	−0.296 **	−0.265 **	0.420 **				
11. INAD	6.26	2.95	−0.080	0.204 **	0.372 **	0.484 **	0.580 **	−0.220 **	−0.281 **	−0.301 **	0.526 **	0.727 **			
12. PC	16.01	4.81	0.060	−0.206 **	−0.199 **	−0.274 **	−0.230 **	0.479 **	0.364 **	0.355 **	−0.269 **	−0.333 **	−0.288 **		
13. TS	15.86	5.58	0.155 **	−0.238 **	−0.298 **	−0.318 **	−0.285 **	0.354 **	0.571 **	0.425 **	−0.359 **	−0.322 **	−0.300 **	0.665 **	
14. RAP	11.15	3.78	0.042	−0.170 **	−0.183 **	−0.274 **	−0.239 **	0.378 **	0.462 **	0.473 **	−0.202 **	−0.288 **	−0.259 **	0.704 **	0.798 **

Note. * *p* < 0.05 (2-tailed); ** *p* < 0.01 (2-tailed), SEX: 0 = Female, 1 = Male, EXH = Emotional exhaustion, CYN = Cynicism, INAD = Sense of inadequacy, PC = Positive climate, TS = Teacher sensitivity, RAP = Regard for adolescent perspective.

**Table 2:** Hierarchical multiple regression analyses of perceived teacher emotional support dimensions at T1 on burnout dimensions at T2.

Variables	Perceived Teachers’ Emotional Support Dimensions at T1 on Emotional Exhaustion at T2			Perceived Teachers’ Emotional Support Dimensions at T1 on Cynicism at T2			Perceived Teachers’ Emotional Support Dimensions at T1 on Sense of Inadequacy at T2		
	β	t	Model Summary	β	t	Model Summary	β	t	Model Summary
Step 1			Adj. R^2^ = 0.43 **			Adj. R^2^ = 0.41 **			Adj. R^2^ = 0.33 **
AGE	0.07	1.54	F(2, 292) = 112.71	0.09	1.95	F(2, 292) = 103.03	0.08	1.84	F(2, 292) = 76.55
EXH1	0.63	14.10 **							
CYN1				0.61	13.43 **				
INAD1							0.56	11.60 **	
Step 2			Adj. R^2^ = 0.45 **			Adj. R^2^ = 0.41			Adj. R^2^ = 0.34
PC	0.04	0.73	F(3, 289) = 5.17	−0.07	−1.27	F(3, 289) = 1.38	−0.00	−0.15	F(3, 289) = 2.12
TS	−0.25	−3.82 **	ΔR = 0.029	−0.02	−0.40	ΔR = 0.008	0.05	0.73	ΔR = 0.014
RAP	0.09	1.55		−0.02	−0.31		−0.15	−2.25 *	

Note. ** *p* < 0.01. * *p* < 0.05. Age and the corresponding outcome at T1 were controlled for each regression model; EXH1 = Emotional exhaustion at T1, CYN1 = Cynicism at T1, INAD1 = Sense of inadequacy at T1, PC = Positive climate. TS = Teacher sensitivity. RAP = Regard for adolescent perspective.

## Data Availability

The data presented in this study are available on request from the corresponding author.
